# Deferoxamine preconditioning ameliorates mechanical ventilation-induced lung injury in rat model via ROS in alveolar macrophages: a randomized controlled study

**DOI:** 10.1186/s12871-018-0576-7

**Published:** 2018-08-18

**Authors:** Weilin Zhu, Yuansi Huang, Yuqiong Ye, Yafeng Wang

**Affiliations:** Department of Anesthesia, People’s Hospital of Guangxi Zhuang Autonomous Region, Nanning, 530021 China

**Keywords:** Ventilator-induced lung injury, Deferoxamine, Reactive oxygen species, Mitochondria, Alveolar macrophages

## Abstract

**Background:**

Mechanical ventilation (MV) can provide effective breathing support; however, ventilatior-induced lung injury (VILI) has also been widely recognized in clinical practice, including in the healthy lung. Unfortunately, the morbidity and mortality of VILI remain unacceptably high, and no satisfactory therapeutic effect can be achieved. The current study aimed to examine the effects of iron chelator preconditioning on the mitochondrial reactive oxygen species (ROS) in alveolar macrophages and pathological lung injury in VILI.

**Methods:**

Twenty four healthy male Sprague–Dawley (SD) rats (250–300 g in weight) were randomly divided into 3 groups, including the control group (NC group, *n* = 8), the high-volume mechanical ventilation group (HV group, *n* = 8), and the deferoxamine treatment group (HV + DFO group, n = 8). Rats in the HV and HV + DFO groups were subjected to high-volume MV at a dose of 40 ml/kg. DFO was administered at a dose of 200 mg/kg 15 min prior to over-ventilation. Spontaneously breathing anesthetized rats were used as the controls. The animals were sacrificed after 4 h of high-volume ventilation or under control conditions, the animals were sacrificed. Purified alveolar macrophages from bronchoalveolar lavage fluid (BALF) and lung tissue were collected for further analysis through light microscopy and flow cytometry.

**Results:**

Compared with the controls, the high-volume-ventilated rats had exhibited typical lung edema and histological lung injury, and ROS were markedly increased in alveolar macrophages and mitochondria. Moreover, all indices of VILI were remarkably different in rats treated with DFO preconditioning. DFO could ameliorate lung injury in the mechanically ventilated SD rat model.

**Conclusions:**

DFO preconditioning contributes to mitigating the histological lung damage while reducing ROS levels in alveolar macrophages and mitochondria, suggesting that iron metabolism in alveolar macrophages may participate in VILI.

Mechanical ventilation (MV) is necessary for patients under anesthesia during surgery, which can provide respiratory support for the whole body. It is also used to treat patients with acute lung injury (ALI), acute respiratory distress syndrome (ARDS) and other diseases. MV can provide effective breathing support; nonetheless, VILI has also been widely recognized in clinical practice [1, 2].

At present, biological lung injury is known to be associated with the release of inflammatory factors, apoptosis, endothelial injury, and activation of the coagulation system [3, 4]. However, the existing therapeutic effect is limited targeting the above mechanisms. Consequently, it is important to explore new treatment ideas and mechanisms. Alveolar macrophages, which reside in the alveolar space, have accounted for 90% of all leukocytes, and the remainders are mainly dendritic cells and T cells. Alveolar macrophages (AMs) play a crucial role in maintaining immunological homeostasis and host defense, which are also the primary producers of pro-inflammatory cytokines in lung following exposure to noxious stimuli [5, 6]. Oxidative stress in mitochondria can promote the pathology of degenerative diseases and tumorigenesis [7]. Mitochondrion is the main organelle involved in the action of AMs, and also the key initiating factor of the pulmonary inflammatory reaction in early ALI [8]. Previous data reveal that AMs exposed to asbestos fibers can produce H_2_O_2_. In addition, the production of reactive oxygen species (ROS) can be reduced by knocking down the iron-sulfur protein of complex III in the mitochondrial electron transport chain (ETC), a major site responsible for ROS production [9]. Lung diseases can be treated through targeting oxidative stress with antioxidant agents, such as thiol molecules, polyphenols and superoxide dismutase (SOD) [10]. Moreover, the introduction of low tidal volume ventilation has improved the clinical outcome [11]**.**

However, the morbidity and mortality of VILI remain unacceptably high at present; besides, the therapeutic effect is limited for the previously discovered mechanisms. Unfortunately, no preventive therapeutic agent is available currently. Iron chelators are the important antioxidant drugs [12, 13]. There is currently no more effective method to change the ROS levels in the mitochondria of AMs in VILI, and whether an iron chelator is effective in lung MV remains to be further studied.

The current study aimed to explore whether the iron chelator deferoxamine (DFO) had a lung protective effect against VILI in rats and to preliminarily extract a potential new mechanism of MV-induced lung injury.

## Materials and methods

### Animals

The Sprague-Dawley (SD) rat model of VILI was established as previously described [14]. Healthy male adult SD rats weighing 250-300 g were provided by the Laboratory Animal Center of Guangxi Medical University (Nanning, China, License number: SCXK GUI 2009–0002), and raised in an environmentally controlled room at 23 ± 2 °C and 50–70% humidity under a 12/12 h light/dark cycle. All animals were allowed free access to food and water.

The study was performed in accordance with the guidelines for the Care and Use of Laboratory Animals approved by Ethics and Animal Welfare Committee of the People’s Hospital of Guangxi Zhuang Autonomous Region (Nanning, China, Permission number 2014–034). All possible efforts were undertaken to avoid animal suffering at each stage of the experiments.

### Establishment of the VILI rat model

16 male SD rats were randomly divided into two groups, including the control group (group NC) and lung injury group (group HV) Group NC received an intraperitoneal injection of 10% chloral hydrate (1.5 mL/kg) to induce anesthesia. The rats were intubated with a tracheal cannula after tracheotomy upon the cease of body movement. The animals were fixed in a supine position on the operating table and were performed thoracotomy to remove the lung tissues under aseptic conditions. Group HV underwent an intraperitoneal injection of 10% chloral hydrate (1.5 mL/kg) to induce anesthesia. The animals were also fixed in a supine position on the operating table, intubated with a tracheal cannula after tracheotomy, and fixed with surgical line nodes connected to a small animal ventilator (TOPO, Kent Scientific, Torrington, USA) for MV. The respiratory parameter settings were as follows: a tidal volume of 40 mL/kg, a respiratory frequency of 40–60/min, a respiratory ratio of 1:1, and a positive end-expiratory pressure of 0. All rats were given supplemental oxygen at approximately 40–50%. Blood samples were collected from the anatomical femoral artery indwelling tube for blood gas analysis. The respiratory rate was adjusted using the partial pressure of carbon dioxide in arterial blood (PaCO_2_) and maintained at 35–45 mmHg. After MV for 4 h, lung tissues were collected under sterile conditions. Animals in both groups were sacrificed by bloodletting.

### Hematoxylin and eosin (HE) staining

**Fig. 1:**
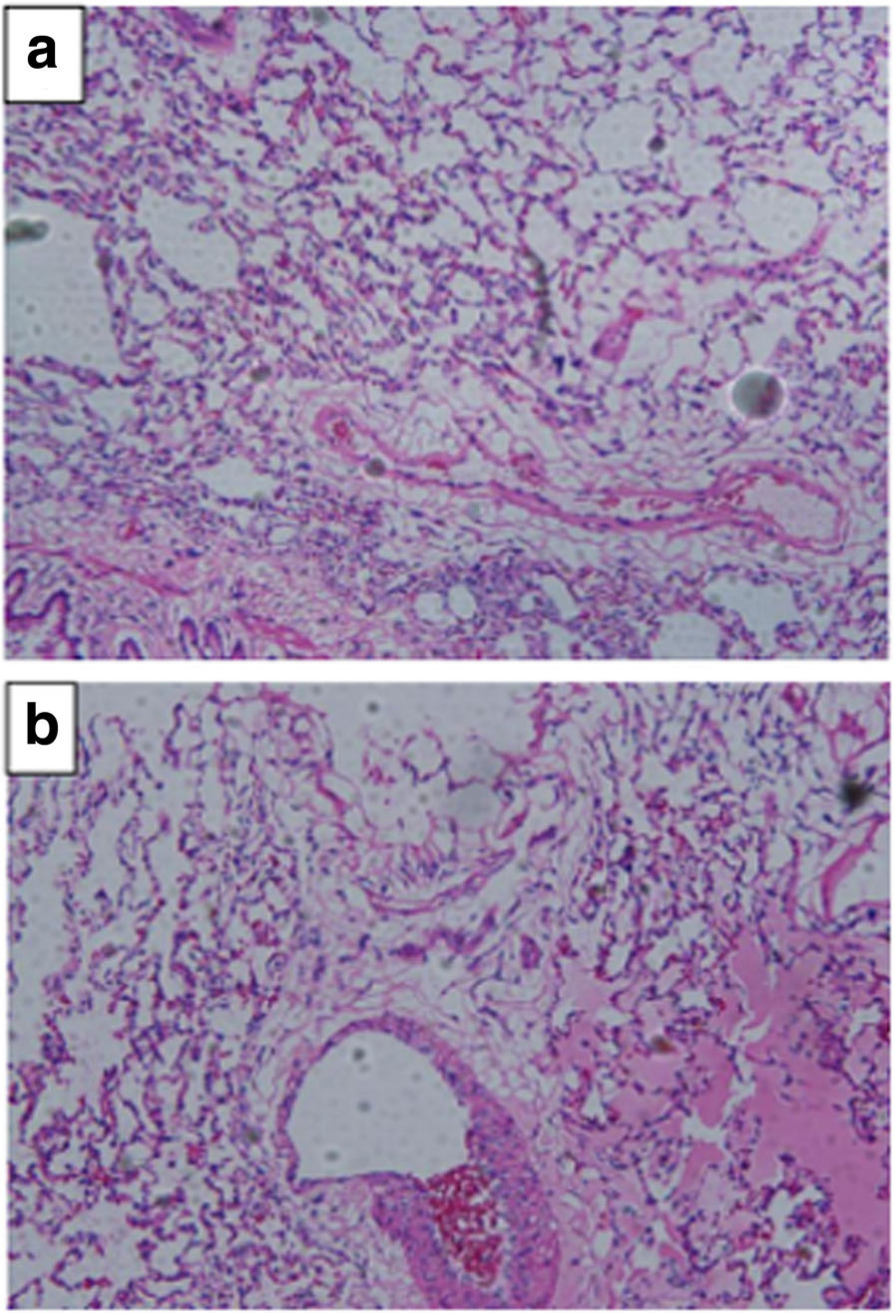
Estblishment of a VILI rat model. **a** Group NC showed lung alveolar structure and clear alveolar exudate, × 100. **b** In group HV, epithelial shedding in the lung alveoli was visible after 4 h of VILI tidal volume of 40 mL/kg, × 100

Tissues from the left lung were cut into 3 blocks 0.1 cm × 0.1 cm × 0.1 cm in size, fixed with 10% formalin, and sent to the Department of Pathology of Guangxi Zhuang Autonomous Region People’s Hospital for pathological sectioning and observation under a light microscope (Olympus, Japan). The pathological observation results showed that the lung injury model had been successfully established by MV with a high tidal volume (tidal volume of 40 mL/kg, respiratory frequency of 40–60/min, and ventilation for 4 h).(Fig. 1).

### Establishment of a MV model with preconditioning

Twenty four healthy adult male SD rats weighing 250–300 g were randomly divided into three groups, with 8 rats in each group. The control group (group NC) underwent intraperitoneal injection of 10% chloral hydrate (1.5 mL/kg) 5 min after tracheal intubation and thoracotomy. The high tidal volume ventilation group (group HV) received intraperitoneal injection of 10% chloral hydrate (1.5 mL/kg). 5 min later, 2 mL saline was injected intraperitoneally. Ventilator-controlled breathing and tracheal intubation were initiated 15 min later based on the following parameters, a tidal volume of 40 mL/kg, a frequency of 40–60/min, a I:E ratio of 1:1, and a positive end-expiratory pressure of 0. All rats were administered supplemental oxygen at approximately 40–50%. Blood samples were collected from the anatomical femoral artery indwelling tube for blood gas analysis. The respiratory rate was adjusted using the partial pressure of carbon dioxide in arterial blood (PaCO_2_) and maintained at 35–45 mmHg. The group DFO (group HV + DFO) was given an intraperitoneal injection of 10% chloral hydrate (1.5 mL/kg), followed by intraperitoneal injection of DFO (200 mg/kg, Sigma, USA) 5 min later. 15 min later, tracheal intubation with a ventilator was performed to control breathing in the same way as that in the group HV. After MV for 4 h, the left lung was ligated, and the lung blood was flushed through the pulmonary artery with solution 2. Animals in the three groups were sacrificed by rapid bloodletting, the whole trachea, heart and lungs were removed, and the corpses were disposed properly.

### Lung wet/dry ratio (W/D)

Part of left lung tissues were collected and weighed on an electronic balance, and the total wet weight was recorded as W. The samples were then incubated at 80 °C in a drying oven for 48 h to obtain the total dry weight, which was recorded as D. Finally, the W/D ratio was calculated to assess pulmonary edema.

### Lung tissue pathological injury

Fresh left lung tissue blocks approximately 0.1 cm × 0.1 cm × 0.1 cm in size were fixed with 10% formaldehyde and sent to the Department of Pathology of Guangxi Zhuang Autonomous Region People’s Hospital for observation. Paraffin sections were produced, stained with HE, observed under a light microscope (Olympus, Japan), and graded according to both histological and morphometric analysis introduced by Smith [15].

### Preparation of the AM suspension solution

The right lung with endotracheal tube was placed into a 50 ml beaker, and washed with 1 x PBS using the transtracheal catheter for 10 times. The lavage fluid was collected and placed into a 50 ml 130 g × 8 min centrifuge tube for centrifugation. The supernatant was collected, and 3 ml red blood cell lysate was added, followed by ice cracking for 10 min. The mixture was sufficiently mixed and centrifuged at 440G for 10 min to collect the supernatant. 2 ml red blood cell lysate was then added to centrifuge at 440G for 10 min, and the supernatant was collected, which was the macrophage suspension. According to related literature, the alveolar lavage fluid mainly consisted of AMs.

Tracheal intubation with lung lavage was performed after washing with 6 mL PBS for 10 times. The lavage fluid was collected and transferred to a 50 mL centrifuge tube for centrifugation (Hettich, Germany) at 4 °C at 130 g for 8 min. The supernatant was discarded, and then 3 ml red blood cell lysate was added, followed by ice cracking for 10 min, centrifugation at 440 g for 10 min, for twice. The precipitate was suspended in PBS, and the cell concentration was adjusted to 2 × 106/mL- 3 × 106/mL. The purity was detected by Giemsa staining, the cell activity was detected by the trypan blue exclusion assay and counted using a hemocytometer.

### Detection of ROS in macrophages

Cytometric detection of ROS in macrophages was conducted through assay (Beyotime Biotechnology, China) by flow cytometry (BD FacsCalibur, USA). Samples loaded with probes were detected by flow cytometry. The excitation and emission wavelengths were 510 nm and 580 nm, respectively, which were used to detect the stimulated fluorescence intensity in a real-time manner.

### Detection of mitochondrial ROS in macrophages

Detection of mitochondrial ROS in macrophages through assay (Life Technologies, USA) by flow cytometry. Alveolar macrophages were suspended in MitoSOXTM liquid by dilution. Samples loaded with probes were detected by flow cytometry. The excitation and emission wavelengths were 510 nm and 580 nm, respectively, which were used to detect the fluorescence intensity in a real-time manner using the Reactive Oxygen Detection Kit (Life Technologies, USA).

### Statistical analysis

Animals were required to obtain a power of 0.80 with a *p* < 0.05 and a SD = 1/2 mean.

Some data had to be excluded due to technical problems; as a result, the subsequent lung analyses were done with 8 samples per group as indicated. SPSS 24 statistical software was employed to determine the mean ± SD and perform single factor analysis of variance. Intracellular and mitochondrial ROS were analyzed using the Flowjo 7.6.2 software (BD, USA). Data were compared using the chi-squared test or Fisher’s test, and differences with a value of *p* < 0.05 were considered statistically significant.

## Results

### Lung W/D ratio

**Fig. 2:**
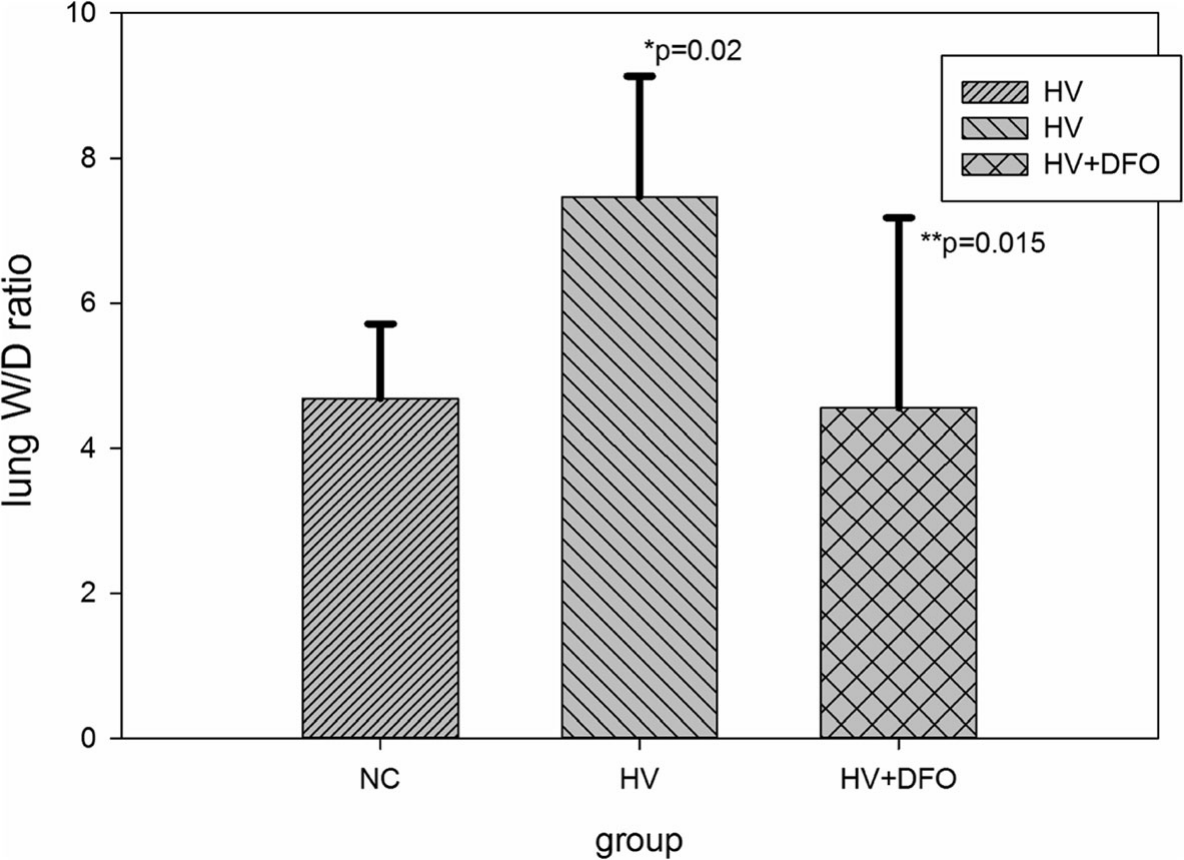
Comparison of lung W/D ratio among the three groups ((Mean ± SD). Part of the left lung tissues were collected and weighed on an electronic balance, and the total wet weight was recorded as W. The samples were then incubated at 80 °C in a drying oven for 48 h to obtain the total dry weight, which was recorded as D. The value of W/D ratio was calculated to assess pulmonary edema. *p* = 0.054 by Levene statistics; F = 53.39 and *p* = 0.000 between groups. *p = 0.02 (95%CI 4.01, 5.17), compared with group NC, and **p = 0.015(95%CI -5.30,-0.53) compared with group HV by Tukey HSD statistics

The W/D ratio was markedly increased in group HV compared with that in group NC group (*p* = 0.02). In the meantime, the W/D ratio was outstandingly decreased in group HV + DFO relative to that in group HV (*p* = 0.015) (Fig. 2).

### Lung tissue pathological damage under a light microscope

**Fig. 3:**
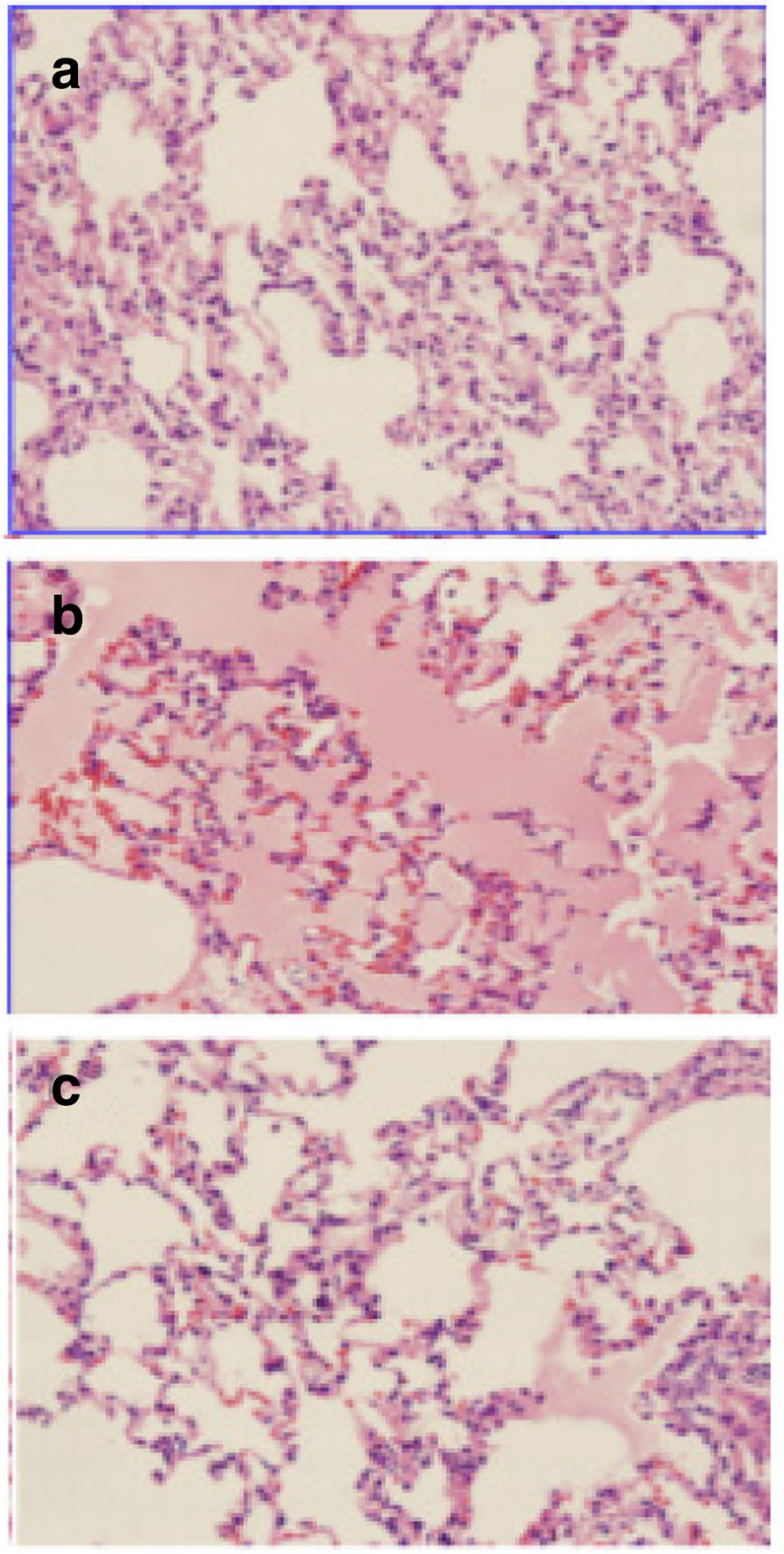
Pathological sections of lung tissue were observed under a light microscope. **a** Normal lung tissue morphology, with no protein exudation in alveolar cavity and no alveolar epithelium peeling (group NC, × 200). **b** Visible alveoli with fibrin exudation, and the epithelial cavity was visible (group HV, × 200). **c** Small amount of alveolar epithelial shedding, along with normal lung interval (group HV + DFO, × 200)

In group NC, the lung tissue was clear, the alveoli were intact and free of congestion and edema, and no inflammatory exudation was present. In comparison, the lung tissue in group HV showed inflammatory cell infiltration. Group HV + DFO displayed mild pathological changes, which mainly manifested as capillary congestion, alveolar fibrin with little exudation, slight alveolar septum widening, occasional exfoliated epithelial cells in the lumen, as well as rare alveolar and interval neutrophilic exudation (Fig. 3).

### Lung injury score by both histological and morphometric analysis

**Fig. 4:**
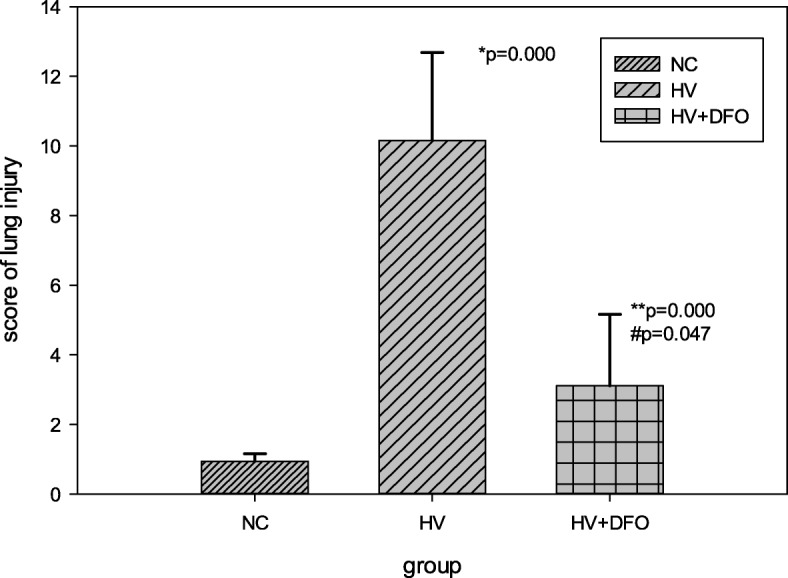
Both histological and morphometric analysis of lung injury among the three groups ((Mean ± SD). *p* = 0.001 by Levene statistics; Welch statistic F = 53,39, and *p* = 0.000 between groups. *p = 0.000 (95%CI 11.85, 6.59), ^#^*p* = 0.047(95% CI 0.37, 4.30) compared with group NC, and **p = 0.000 (95% CI -10.07, − 4.03) compared with group HV by Games-Howell statistics

Compared with group NC, the he lung injury score was markedly increased in group HV (*p* = 0.000). In group HV + DFO, the lung injury score (*p* = 0.047) was increased to a certain extent, but was apparently lower than that in group HV (*p* = 0.000) (Fig. 4).

### The purity and activity of alveolar macrophages

Macrophage suspensions were prepared and purified using BALF. The purity was detected through Giemsa staining. The cell concentration was adjusted to 2 × 10^6^/mL- 3 × 10^6^/mL. The cell activity was detected by trypan blue exclusion assay and counted using a hemocytometer. According to the trypan blue exclusion assay, the cell survival rate was as follows, group NC: 90.42 ± 3.70%, group HV: 88.37 ± 5.21%, and group HV + DFO:87.00 ± 4.83% (F = 0.79,*p* = 0.467). Results of Giemsa staining and cell suspensions by flow cytometry analysis suggested that the cell liquid purity rates were as follows, group NC: 90.87 ± 4.36%, group HV: 86.50 ± 6.00%, and group HV + DFO: 87.75 ± 8.15% (F = 1.03,*p* = 0.384).

### Determination of ROS in cells and macrophage mitochondria

**Fig. 5:**
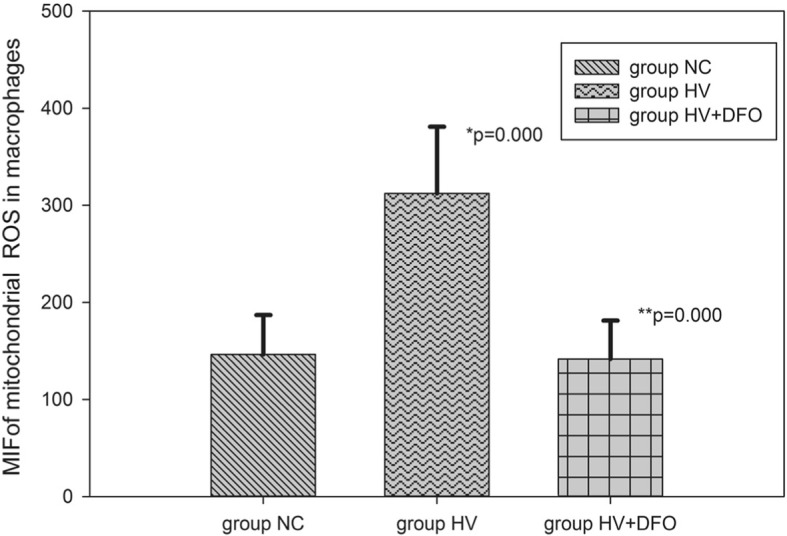
The mean fluorescence intensity (MFI) of mitochondrial ROS in AMs among the three groups ((Mean ± SD). *p = 0.03 compared with group NC, and **p = 0.017 compared with group HV

**Fig. 6:**
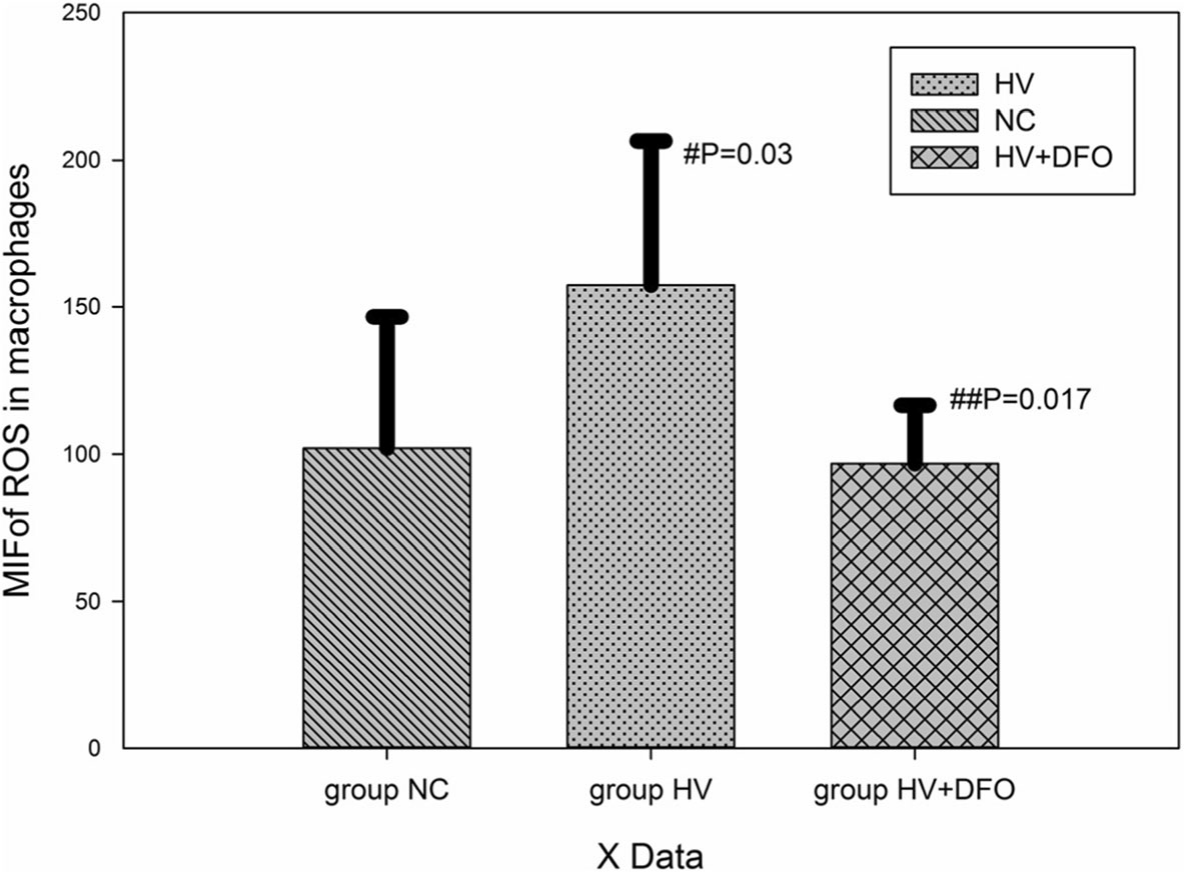
Comparison of MFI of ROS in AMs among the three groups ((Mean ± SD). ^#^ *p* = 0.03 compared with group NC, and ^##^*p* = 0.017 compared with group HV

**Fig. 7:**
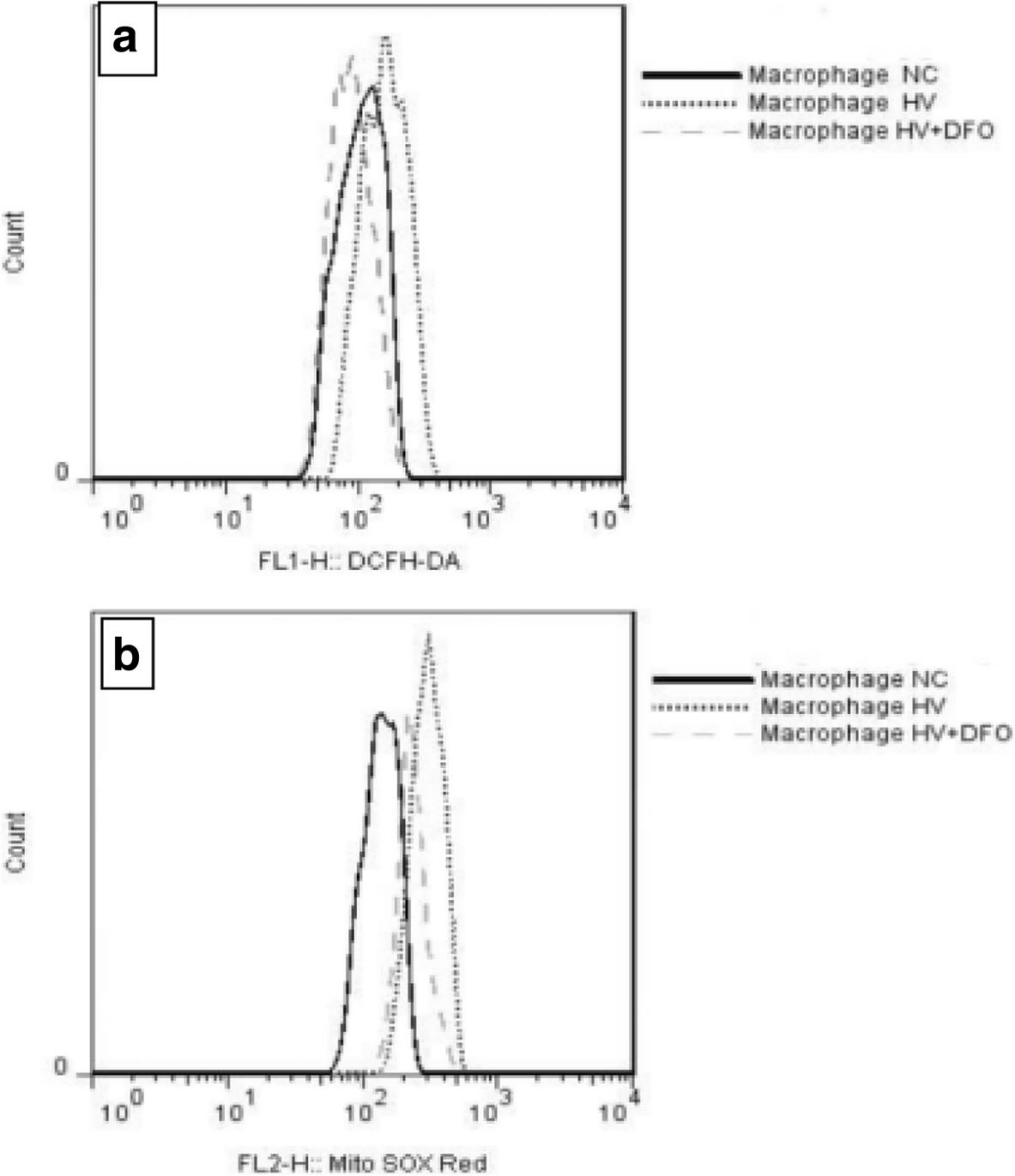
**a** MFI of ROS in macrophages. Compared with group NC, the peak deviation of group HV was shifted to the right, and the peak left deviation of group HV + DFO was similar to that of group NC. **b** MFI of ROS in macrophage mitochondria by flow cytometry. Compared with group NC, the peak deviation of group HV was shifted to the right, and the peak left deviation of group HV + DFO was similar to that of group NC

Results of flow cytometry showed that the mean fluorescence intensity (MFI) of ROS in cells and macrophage mitochondria in group HV was dramatically increased compared with that in group NC (*p* = 0.03,*p* = 0.000). In contrast, the MFI in cells and mitochondria was notably decreased in group HV + DFO compared with that in group HV (*p* = 0.017,*p* = 0.000) (Figs. 5, 6, 7).

## Discussion

The important findings of this study are that VILI in AMs and mitochondrion-released ROS are involved in the injury process, while DFO preconditioning can outstandingly reduce ROS release from AMs and mitochondria, mitigate pulmonary edema, alleviate pathological damage, and show lung protection.

MV, which can provide effective respiratory support, can also lead to severe lung injury known as VILI. An increasing number of studies have been conducted to examine the inflammatory factors and cells involved in lung injury, which suggest that macrophages are important cells involved in the pathogenesis of VILI [5, 6], and the main cells participating in the inflammatory response [16].

Additionally, relevant literature shows that the BALF cells mainly consist of AMs [17], which conforms to our results. Recent studies have found that oxidative stress is involved in mechanical injury [18–20], and ROS production is increased in many cells with increased tensile stress [21–23]. ROS is an important target of VILI, as discovered from ROS generated by antioxidant treatment [24, 25]. However, the origin and possible mechanism of ROS production remain unclear. Mitochondria are the main sites responsible for ROS production [26, 27]. Nonetheless, the role of macrophage mitochondria in MV-induced lung injury remains to be further elaborated. Besides, it has not been reported whether preventive medication can prevent MV-induced lung injury.

Increasing evidence indicates that oxidative stress originating from iron overload plays an important role in various lung diseases, including asthma, chronic obstructive pulmonary disease (COPD), ALI, pulmonary fibrosis and the pathogenesis of lung cancer [28–31]. Chelation of free iron with iron amine has been shown to protect against lung injury caused by oxidative stress in some lung diseases [32, 33].

DFO has a specific hydroxylamine group iron chelator and shows a high affinity for Fe^3+^. Thus, reducing ROS attack generated by iron ions on biofilms is a very effective way to prevent iron ions from being reduced to ferrous ions and participating in oxidative stress. Therefore, iron chelators have gradually become the focus in the tissue protection field. Iron overload will result in increased ROS release in cancer, brain injury and other diseases, giving rise to tissue damage [34–37]. However, it remains unclear whether this reaction is involved in iron overload. Our findings reveal that ROS production in AMs and mitochondria are increased in VILI (*p* < 0.05), and the ROS increasing range in mitochondria is higher than in macrophages. In addition, intracellular or mitochondrial ROS can be reduced by DFO preconditioning (*p* < 0.05). However, DFO is a non-selective mitochondrial antioxidant; consequently, further study is required to determine whether intracellular ROS is derived from mitochondrial deformation. Group HV + DFO has exhibited reduced pathological damage, and the lung W/D ratio is remarkably decreased (*p* < 0.05), which is consistent with the ROS production trend observed in macrophages and mitochondria. Such result suggests that prophylactic intraperitoneal injection of the iron chelator DFO can effectively reduce ROS formation in macrophages and mitochondria in SD rats. Additionally, it can also inhibit the oxidative damage to biological membranes, and reduce pulmonary edema as well as lung pathological damage. Such finding indicates that iron overload may be involved in VILI. The current study is inevitably associated with certain limitations. Specifically, DFO displays no specificity in inhibiting mitochondrial ROS production; therefore, it can not be ensured that ROS originate in mitochondria. On this account, our next step will be to further study the effects of the mitochondrion specific iron chelator on MV-induced lung injury, and the role of DFO preconditioning in hyperoxic alveolar damage.

In conclusion, preconditioning with intraperitoneal injection of the iron chelator DFO can effectively reduce the ROS production in macrophages and mitochondria, as shown in the MV-induced lung injury SD rat model, resulting in reduced pathological damage and showing lung protection. It is also suggested that oxidative stress induced by iron overload in macrophages may be involved in VILI, which should be further verified in studies.

## Conclusions

DFO preconditioning contributes to mitigating the histological lung damage while reducing ROS levels in alveolar macrophages and mitochondria, suggesting that iron metabolism in alveolar macrophages may participate in VILI.
